# The effectiveness of critical time intervention for abused women leaving women’s shelters: a randomized controlled trial

**DOI:** 10.1007/s00038-017-1067-1

**Published:** 2018-01-03

**Authors:** Danielle A. M. Lako, Mariëlle D. Beijersbergen, Irene E. Jonker, Renée de Vet, Daniel B. Herman, Albert M. van Hemert, Judith R. L. M. Wolf

**Affiliations:** 1 Impuls - Netherlands Center for Social Care Research, Department of Primary and Community Care, Radboud university medical center, Nijmegen, The Netherlands; 2 Amsterdam University of Applied Sciences, Amsterdam, The Netherlands; 3 Silberman School of Social Work at Hunter College, City University of New York, New York, NY USA; 4 Department of Psychiatry, Leiden University Medical Center, Leiden, The Netherlands

**Keywords:** Intimate partner violence, Abused women, Women’s shelters, Intervention, RCT, Quality of life

## Abstract

**Objectives:**

To examine the effectiveness of critical time intervention (CTI)—an evidence-based intervention—for abused women transitioning from women’s shelters to community living.

**Methods:**

A randomized controlled trial was conducted in nine women’s shelters across the Netherlands. 136 women were assigned to CTI (*n* = 70) or care-as-usual (*n* = 66). Data were analyzed using intention-to-treat three-level mixed-effects models.

**Results:**

Women in the CTI group had significant fewer symptoms of post-traumatic stress (secondary outcome) (adjusted mean difference − 7.27, 95% CI − 14.31 to − 0.22) and a significant fourfold reduction in unmet care needs (intermediate outcome) (95% CI 0.06–0.94) compared to women in the care-as-usual group. No differences were found for quality of life (primary outcome), re-abuse, symptoms of depression, psychological distress, self-esteem (secondary outcomes), family support, and social support (intermediate outcomes).

**Conclusions:**

This study shows that CTI is effective in a population of abused women in terms of a reduction of post-traumatic stress symptoms and unmet care needs. Because follow-up ended after the prescribed intervention period, further research is needed to determine the full long-term effects of CTI in this population.

**Electronic supplementary material:**

The online version of this article (10.1007/s00038-017-1067-1) contains supplementary material, which is available to authorized users.

## Introduction

Intimate partner violence (IPV) is a major global health burden and is committed mostly against women (World Health Organization 2012). More than 50,000 abused women and their children sought refuge in women’s shelters to escape violence on any day in 2014–2015, within the Global Network of Women’s Shelters (GNWS 2015). Shelter-based abused women generally report more severe abuse and related injury (Saunders 1994) and are at a higher risk for post-traumatic stress disorder (PTSD) (Jones et al. 2001) than other abused women. Because they often have few resources in terms of education, income, work, and social support, they have to rely on women’s shelters for support (Cense et al. 2006; Wittebrood and Veldheer 2005).

After shelter discharge, abused women continue to have a need for follow-up support services (Ham-Rowbottom et al. 2005; Wolf et al. 2007). A lack of resources—such as income and social support—makes it difficult to build a stable life in the community (Sullivan et al. 1992; Wolf et al. 2007). Moreover, during the transition from shelter to community living, women are at great risk to experience re-abuse by their ex-partner (Fleury et al. 2000).

Women who obtain desired resources and are able to increase their social support, experience improved quality of life (QoL), which in turn appears to lower their risk for re-abuse (Bybee and Sullivan 2002). However, research into interventions focusing on this transition phase is scarce (Jonker et al. 2015; Rivas et al. 2015). To our knowledge, only one longitudinal randomized controlled trial (RCT) primarily focused on abused women has been conducted. Sullivan and Bybee (1999) examined the effectiveness of a 10-week advocacy intervention for IPV victims after shelter discharge. Two years after the intervention ended, women who had worked with advocates reported higher QoL and social support, were more effective in obtaining resources, and had a lower risk for re-abuse than women who did not.

Critical time intervention (CTI) is a time-limited, strengths-based intervention designed to support vulnerable people during transitions in their lives. The intervention’s goals are to expand clients’ networks, involve their social and professional networks, and reassure continuity of care and support during the transition. Furthermore, the case managers trained in CTI provide practical (helping out with, e.g., home furnishing and finding daily activities) and emotional support during the transition. In the US, CTI has been found effective in reducing recurrent homelessness and psychiatric symptoms in homeless men with severe mental illness (Herman et al. 2000; Susser et al. 1997) and has been adapted for other populations such as homeless families (Shinn et al. 2015). So far, the effectiveness of CTI for abused women has not yet been examined. Because the goals of CTI strongly resemble the needs of abused women leaving shelters we identified CTI as a potentially effective intervention. Furthermore, this study may show that CTI is also effective outside the US in a different health care system.

This study investigates the effectiveness of CTI for abused women transitioning from women’s shelters to community living in an RCT. We hypothesized that CTI is more effective than care-as-usual in: (1) improving QoL (primary outcome); (2) preventing re-abuse, reducing symptoms of depression, PTSD, and psychological distress, improving self-esteem (secondary outcomes); and (3) improving family support, social support, and the fulfillment of care needs (intermediate outcomes). We performed a fidelity assessment with a subsample of 35 women assigned to CTI (de Vet et al. 2017b).

## Methods

### Study design and setting

This study was a multicenter, parallel-group RCT. Women were recruited from women’s shelter organizations which were part of the Academic Collaborative Center for Shelter and Recovery in 2009. Eligible shelters provided 24-h services with a duration of at least 6 weeks, served at least 50 unique adult women per year, and expected to continue services over the next 5 years. Nine out of 12 participating organizations in the Academic Collaborative Center for Shelter and Recovery with 19 shelter facilities were selected to participate. The Medical Review Ethics Committee region Arnhem-Nijmegen declared that the study was exempt from formal review (registration number 2011/038). The study is registered at the Netherlands Trial Register (NTR3463). The study protocol is described in detail elsewhere (Lako et al. 2013).

### Participants and procedures

Women were eligible if they: (1) were aged ≥ 18; (2) stayed at the shelter due to IPV or honor-related violence (violence committed to restore or prevent violation of the family honor^1^); (3) stayed at the shelter for ≥ 6 weeks; (4) had a set date of departure from the shelter or received priority status for social housing, and (5) were moving to housing without daily supervision or support where they would have to pay rent or housing costs. Women could not participate if they were moving to a region where none of the participating organizations provided services.

Recruitment occurred from December 2010 to July 2012. At each shelter one staff member screened women for eligibility. If women were eligible and expressed interest in participation, they were asked for permission to provide their contact information to the research team. A research assistant scheduled the baseline interview (T0). After complete description of the study to the women, written informed consent was obtained. The baseline interview was structured and administrated face-to-face. Follow-up interviews were conducted by telephone at 3 months (T3) and 6 months (T6) and face-to-face at 9 months (T9). Women who did not speak Dutch were interviewed by multilingual research assistants or research assistants who were assisted by professional interpreters by translating the interview via telephone (T0: 24%; T9: 23%). Because the translation would considerably lengthen these interviews, a shortened version of the questionnaire was used (not pre-specified in the protocol).

### Randomization and blinding

A researcher (D.L.) generated the randomization sequence using a computer random number generator. Randomization was stratified by shelter with a 1:1 allocation in blocks of four. The numbers were saved in a secured digital file and concealed until assignment. After T0, one of the researchers (D.L. or R.V.) ascertained condition assignment and informed shelter staff. Shelter staff and the research assistant who scheduled the baseline interviews were unaware of condition assignments in advance. Women were unaware of condition assignment until they met their CTI worker/case manager after T0. Although information about condition assignment was withheld from the research assistants who conducted the follow-up interviews, some of them became aware of the assigned condition of a few women because the women told them about the services received.

### Intervention and control groups

Women in the experimental condition received CTI, which consists of three phases: (1) transition to the community, (2) try-out, and (3) transfer of care (Table 1). In this study, the duration of each phase was predetermined at 3 months. More information about CTI is provided in the study protocol (Lako et al. 2013). Shelter organizations selected two or three case managers to deliver CTI (See Online Resource for more information about CTI training). Before the start of the study, shelter services were professionalized by implementing a strengths-based approach, which focuses on women’s strengths rather than deficits and views the community as a source of opportunities and resources (Wolf and Jansen 2011). Principles from the strengths model (Rapp and Goscha 2006) were integrated in CTI to ensure continuity in service approach during the transition. We conducted a fidelity assessment for a representative subsample of 35 women assigned to CTI and reported the results in previous work (de Vet et al. 2017b). Fidelity was measured using the CTI fidelity scale, a quantitative tool developed by Conover and Herman (2007, unpublished manuscript). The scale items are rated on a 5-point scale ranging from not implemented (1) to ideally implemented (5). The intervention in this study obtained a fidelity score of 3, indicating that CTI was fairly implemented (de Vet et al. 2017b; Conover 2012, unpublished manuscript).

**Table 1:** Components of critical time intervention (CTI) in each phase (The Netherlands, 2010–2013)

Phase	Pre-CTI	Phase 1: transition to the community	Phase 2: try-out	Phase 3: transfer of care
Timing	Between assignment and discharge	Between discharge and 3 months after discharge	Between 3 and 6 months after discharge	Between 6 and 9 months after discharge
Responsibilities of CTI worker	Build a relationship	Build a relationship by working in the community Assess client’s needs and resources Choose priority areas of intervention Mobilize support resources and link client to them	Less frequent contact Adapt, improve and monitor resources	Adapt, improve and monitor resources Transfer client to other services Farewell and termination
Materials^a^	*Required:* Intake form Activity log *Optional:* Strengths assessment Personal recovery plan	*Required:* Risk and needs assessment Strengths assessment Personal recovery plan Activity log	*Required:* Personal recovery plan Activity log *Optional:* Strengths assessment	*Required:* Personal recovery plan Activity log closing note *Optional:* Risk and needs assessment Strengths assessment
Intensity	At least 3 meetings with client and her children (if any) before discharge, with no more than 1 month between each meeting (10 h in total)	Average of 3 h per week (36 h in total)	Average of 2 h per week (24 h in total)	Average of 0.5–1 h per week (6–12 h in total)

A similar table is provided in an article by de Vet et al. (2017a)

^a^A detailed description of the materials can be found elsewhere (de Vet et al. 2017b)

Women in the control condition received care-as-usual. All organizations provided services after discharge, except for one which referred women returning to their (ex-)partner to other services. Most organizations provided support during regular meetings (range of average intensity: 1–3 h per week). The average duration of these services varied widely between organizations (range: 13–52 weeks). All organizations employed a strengths-based approach.

To determine how services in the groups differed, several process measures representing key components of CTI were administrated at T3, T6, and T9 (see Online Resource for detailed information about the key components and process measures).

### Sample size

The required sample size was based on the expected improvement in the QoL score resulting from CTI. The only available information about the improvement in QoL came from Sullivan and Bybee’s (1999) study on advocacy services for abused women leaving shelters. A small but statistically significant difference (effect size of 0.2) was found on a similar scale. A larger improvement in the QoL was expected in this study because the duration and intensity of CTI is much greater than in the advocacy intervention.

To compute the required sample size, a clinically relevant effect size of 0.55 was assumed, constituting a moderate effect, resulting in a mean improvement in the QoL score of 0.8. Each group should contain 55 women to detect this difference with 80% power (*α* = 0.05, two-sided). A potential loss of power was considered due to the clustering of women within CTI workers/case managers. Assuming an intra-class correlation coefficient of 0.05 and an average number of three women per CTI worker/case manager, the number of women in both groups should be increased to 61. Accounting for 30% attrition, the intended sample size was 174. Because the attrition rate was lower (6%) than expected, we ended the recruitment period 1 month earlier than planned.

### Outcomes

All outcomes were assessed at T0 and T9 with the exception of the outcome re-abuse, which was measured at T3, T6, and T9. A complete description of the outcome measures can be found in the protocol (Lako et al. 2013). We decided not to report six outcome measures in this work for several reasons, such as missing data (change in protocol; see Online Resource for more information about the excluded outcomes). However, we analyzed and reported these outcomes to the funding bodies (available on request).

### Primary outcome

QoL was assessed with the average score of two identical items rating general life satisfaction from Lehman’s Brief Quality of Life Interview (Lehman 1983). We asked the question *How do you feel about your life in general?* twice during each interview (at the beginning and at the end). Women answered on a 7-point scale from terrible (1) to delighted (7). Cronbach’s alpha was 0.61 and 0.80, respectively.

### Secondary outcomes

Re-abuse was assessed by the question *Have you been abused since the last interview?* Answer categories were *yes* and *no.* The sum score of the 20-item Center for Epidemiological Studies Depression scale (Radloff 1977) was used to measure depressive symptoms. PTSD symptoms were measured by the sum score of the 15-item Impact of Event Scale (Horowitz et al. 1979). The Global Severity Index, an average score of the 53-item Brief Symptom Inventory (Derogatis 1975) was used to assess psychological distress. The sum score of the 10-item Rosenberg Self-Esteem Scale (Rosenberg 1965) was used to assess self-esteem.

### Intermediate outcomes

So far, the mechanisms through which CTI operates have not been extensively studied (Tomita and Herman 2012). In a study about the effectiveness of CTI on psychiatric rehospitalization among homeless people with severe mental illness the authors wrote “[…] it appears that the effect was not entirely explained by increased housing stability [which was a confounder in the study]. Other possible mechanisms include direct emotional and practical support provided by the CTI worker, enhanced access to treatment services and community supports, as well as reduced psychiatric symptoms” (Tomita and Herman 2012). Therefore, we decided to assess family support, social support and unmet care needs as intermediate outcomes. Family and social support were measured with the average score of five items used in the RAND Course of Homelessness Study (Burnam and Koegel 1989). Women had to indicate on a 5-point scale how often different kinds of support were available to them from family, friends or other acquaintances. Unmet care needs were assessed by asking women whether they wanted and received help in six life domains (e.g., safety and protection against violence). Response categories were adopted from the short form Quality of Life and Care (Wennink and van Wijngaarden 2004). Women were considered to have unmet care needs if they wanted help, but did not receive help in at least one life domain.

### Statistical analysis

All analyses were conducted with SPSS Version 20, using an intent-to-treat approach. Three-level mixed-effects models were used to determine the effectiveness of CTI. Because the number of women per case manager was low (primarily 1:1), we adjusted for clustering within organizations instead of within case managers.

Sociodemographic characteristics and baseline measurements of other outcomes were considered potential covariates unless outcomes were expected to correlate strongly based on shared content (e.g., depression and psychological distress). We inspected the strength of the relationship between these variables and follow-up measurements of outcomes with Pearson’s *r, φ,* or *η*. None of the variables correlated strongly (< 0.5). If the relationship strength was intermediate (0.1 < *x* ≤ 0.5), we inspected for covariate imbalance between groups. If this was detected, the covariate was added to the model to see if model precision improved. None of the covariates yielded a significant improvement on any of the models.

Due to the use of a shortened questionnaire for non-Dutch-speaking women, missing data were higher among this group. This resulted in relevant differences in the distribution (> 10 percentage points) of Dutch-speaking and non-Dutch-speaking women between the T0 and T9 sample in the control group and in the experimental group for several outcomes. To examine whether changes in the distribution of Dutch-speaking and non-Dutch-speaking women would lead to other conclusions regarding the effects, we inspected the scores of both Dutch-speaking and non-Dutch-speaking women in the experimental and control group of the T0 and T9 sample for the outcomes on which we found an effect of CTI (not pre-specified in the protocol). More information about missing data is provided in the Online Resource.

## Results

### Participant flow

**Fig. 1:**
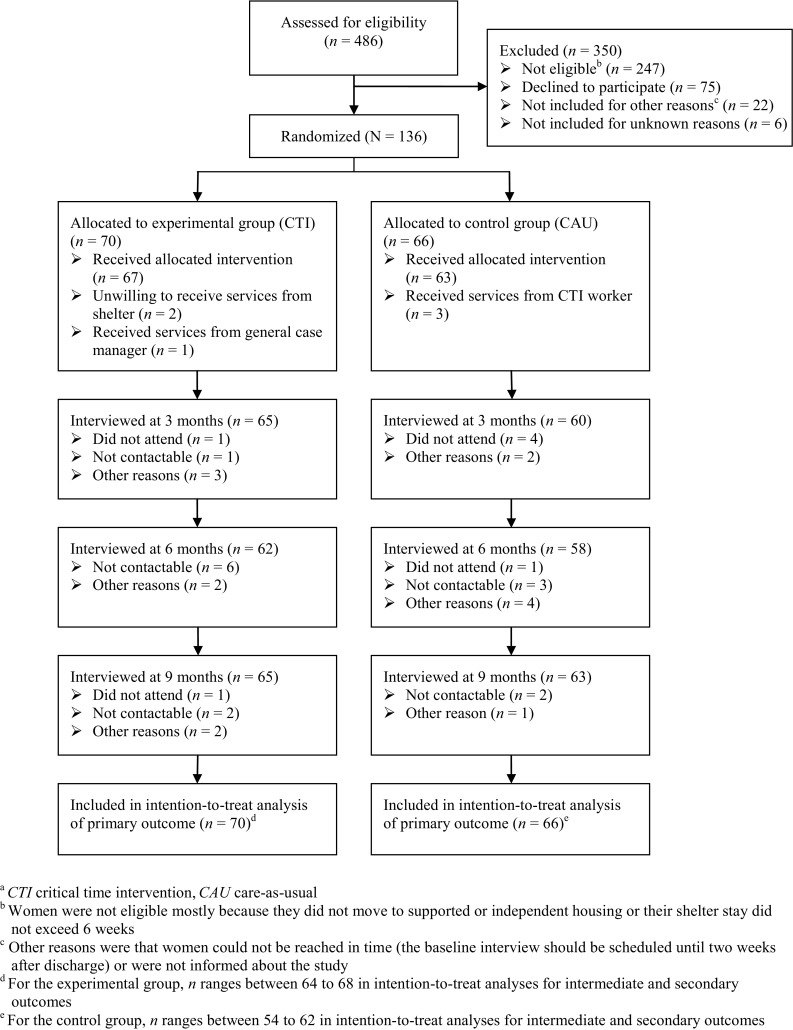
Flow chart of participants in the study into the effectiveness of critical time intervention for abused women leaving women’s shelters (The Netherlands, 2010–2013)^a^

Of 486 women assessed for eligibility, 239 (49%) women were eligible. 136 women were randomly assigned to CTI or care-as-usual (Fig. 1). Non-response analyses comparing participants and non-respondents on country of birth and age showed no significant differences.

### Baseline characteristics

Sociodemographic characteristics and outcomes at baseline are summarized in Table 2. The control group contained more first-generation migrants and more married women. In the experimental group, more women experienced sexual violence. Outcome measures showed some differences between groups at baseline: women assigned to CTI reported fewer symptoms of depression and psychological distress but more symptoms of PTSD than women assigned to care-as-usual.

**Table 2:** Baseline characteristics of women in experimental (critical time intervention) and control (care-as-usual) group (The Netherlands, 2010–2013)

Baseline characteristic	CTI (*n* = 70)	CAU (*n* = 66)
	*M* (SD) or *n* (%)	*M* (SD) or *n* (%)
Age, years	34.24 (8.52)	33.58 (8.08)
Foreign background		
Dutch native	21 (30%)	15 (23%)
First-generation migrant^a^	39 (56%)	43 (65%)
Second-generation migrant	10 (14%)	8 (12%)
Marital status		
Married or registered partnership	22 (31%)	28 (42%)
Divorced or never married	48 (69%)	38 (58%)
One or more children	63 (90%)	59 (89%)
Education level^b^		
Low	47 (68%)	41 (63%)
Intermediate	16 (23%)	14 (22%)
High	6 (9%)	10 (15%)
Violence prior to shelter		
Emotional	68 (100%)	62 (98%)
Physical	52 (77%)	52 (83%)
Sexual	30 (44%)	17 (27%)
Duration of violence, years		
≤ 1	6 (9%)	10 (16%)
1–5	34 (51%)	30 (48%)
5–10	14 (21%)	13 (21%)
> 10	13 (19%)	9 (15%)
General quality of life^c^	4.85 (1.31)	4.61 (1.18)
CES-D score^g^	16.76 (10.91)	20.36 (11.49)
IES score^h^	33.07 (20.06)	29.90 (23.03)
BSI global severity index^i^	0.78 (0.65)	0.88 (0.66)
RSES score^j^	21.15 (4.98)	20.89 (4.92)
Family support^d^	3.18 (1.34)	3.18 (1.28)
Social support^e^	3.52 (1.07)	3.47 (0.89)
Unmet care needs in one or more life domains^f^	59 (91%)	43 (83%)

*CTI* critical time intervention, *CAU* care-as-usual, *M* mean, *SD* standard deviation, *CES*-*D* Center for Epidemiological Studies Depression scale, *IES* Impact of Event Scale, *BSI* Brief Symptom Inventory, *RSES* Rosenberg Self-Esteem Scale

^a^First-generation migrants were from Morocco (22%), Turkey (15%), Iran (6%), Poland (5%), Surinam (5%) and other countries (11% Western and 37% non-Western). We have no information about how long they had been in the Netherlands

^b^*Low* no education, primary education, preparatory secondary vocational education or senior secondary vocational education (assistant training or basic vocational education); *Intermediate* senior secondary vocational education (professional education or middle-management), general secondary education or pre-university education; *High* higher professional education or university education

^c^*n* = 69 in experimental group and *n* = 64 in control group

^d^*n* = 58 in experimental group and *n* = 43 in control group

^e^*n* = 59 in experimental group and *n* = 43 in control group

^f^*n* = 65 in experimental group and *n* = 52 in control group

^g^*n* = 64 in experimental group and *n* = 50 in control group

^h^*n* = 58 in experimental group and *n* = 46 in control group

^i^*n* = 59 in experimental group and *n* = 45 in control group

^j^*n* = 59 in experimental group and *n* = 47 in control group

### Process measures

At T3, 95% of the women in the experimental group received services from a shelter organization (see Online Resource for the process measures). This percentage remained fairly stable over time. The proportion of women in the control group that received services from a shelter organization was lower at every follow-up measurement: from 86% at T3, the proportion dropped to 61% at T9. These data provide evidence that the groups differed in the expected way: women allocated to CTI stayed in contact substantially more often with a shelter organization during follow-up.

Regarding face-to-face contact, 57% of the women assigned to CTI met their CTI worker weekly during the first 3 months, compared with 38% of the women assigned to care-as-usual who met their case manager weekly. At T6, weekly contact was much lower in both groups, but the difference between groups was quite similar compared to the first 3 months (42 and 24%, respectively). We expected that women in the experimental group would have little face-to-face contact at T9. However, a substantial proportion of the women (28%) still had weekly face-to-face contact at T9. In the control group, services were less intensive at every follow-up, but still 17% of the women had weekly face-to-face contact at T9.

Women in the experimental group mostly met their CTI worker in their own house. Three quarters of the women (75–81%) assigned to care-as-usual mentioned also their own house, while a substantial proportion (14–24%) mentioned the case manager’s office. As the protocol prescribes providing the CTI worker’s support being in the community, these proportions show that the support in the experimental group was community-based indeed.

The groups showed some differences in help from other professionals and service agencies. At T9, 30% of the women assigned to CTI indicated receiving help from other professionals and services agencies ‘Always’, while this was 8% in the women assigned to care-as-usual. Thus, women assigned to CTI seem somewhat more successfully linked to community providers than women assigned to care-as-usual.

### Outcomes measures

There was no significant between-group difference in QoL during follow-up (Table 3). For the secondary outcomes, women in the experimental group experienced significantly less symptoms of PTSD during follow-up (adjusted mean difference − 7.27, 95% CI − 14.31 to − 0.22, *p* = 0.04). No between-group differences were found for the other secondary outcomes. Regarding intermediate outcomes, a significant difference between groups was found for unmet care needs: the adjusted odds ratio for group assignment was 0.23 (95% CI 0.06–0.94, *p* = 0.04), indicating that women in the experimental group had a fourfold reduction in the odds for unmet care needs during follow-up. No differential changes between groups were found for the other intermediate outcomes.

**Table 3:** Results of the intention-to-treat analyses of the primary (quality of life), secondary (re-abuse, symptoms of depression, post-traumatic stress disorder, psychological distress, and self-esteem), and intermediate (family support, social support, and unmet care needs) outcomes (The Netherlands, 2010–2013)

	CTI (*n* = 70)		CAU (*n* = 66)		Adjusted mean difference/OR (95% CI)^a^	*p* value
	*n*	*M (SD)* or *n* (%)	*n*	*M (SD)* or *n* (%)		
Primary outcome at T9						
General quality of life	63	5.15 (1.20)	61	4.76 (1.22)	0.20 (− 0.25 to 0.66)	0.38
Secondary outcomes						
Re-abuse at T3, T6 and T9^b^						
T3	64	11 (17%)	59	8 (14%)	–	
T6	64	14 (22%)	59	9 (15%)	1.24 (0.27–5.65)	0.78
T9	64	7 (11%)	59	9 (15%)	0.44 (0.09–2.21)	0.32
CES-D score at T9	58	18.33 (12.64)	53	17.74 (12.13)	3.85 (− 0.34 to 8.03)	0.07
IES score at T9	57	29.04 (20.31)	50	32.19 (19.07)	− 7.27 (− 14.31 to − 0.22)	0.04
BSI global severity index at T9	58	0.76 (0.69)	51	0.75 (0.69)	0.06 (− 0.16 to 0.27)	0.61
RSES score at T9	58	21.06 (5.07)	51	21.04 (5.14)	− 0.11 (− 2.16 to 1.93)	0.91
Intermediate outcomes at T9						
Family support	57	3.11 (1.24)	50	3.49 (1.34)	− 0.36 (− 0.85 to 0.13)	0.15
Social support	56	3.44 (1.04)	49	3.48 (0.93)	− 0.07 (− 0.48 to 0.34)	0.74
Unmet care needs	59	37 (63%)	52	40 (77%)	0.23 (0.06–0.94)	0.04

*CTI* critical time intervention, *CAU* care-as-usual, *T3* 3-month follow-up, *T6* 6-month follow-up, *T9* 9-month follow-up, *M* mean, *SD* standard deviation, *OR* odds ratio, *CI* confidence interval, *CES*-*D* Center for Epidemiological Studies Depression scale, *IES* Impact of Event Scale, *BSI* Brief Symptom Inventory, *RSES* Rosenberg Self-Esteem Scale

^a^ Intention-to-treat analysis for secondary outcome re-abuse adjusted for organization. Intention-to-treat analyses for other outcomes adjusted for baseline scores/proportions and organization

^b^ T3 was used as a reference category

Further inspection of the scores of the Dutch-speaking and non-Dutch-speaking women separately on the significant outcomes of CTI in this study revealed that regarding PTSD, the effect of CTI seems to be based mostly on the decrease in symptoms of non-Dutch-speaking women in the experimental group. With regard to unmet care needs, the effect of CTI seems mostly the consequence of fewer Dutch-speaking women in the experimental group with unmet care needs during follow-up: the proportion of Dutch-speaking women with unmet care needs declined from 88 to 57%, while the proportion of non-Dutch-speaking women with unmet care needs declined from 100 to 90%.

## Discussion

We found evidence for the effectiveness of CTI in reducing the symptoms of PTSD and unmet care needs. However, we did not find effects on the primary outcome, QoL. The finding on PTSD symptoms allows us to extend the existing evidence of the positive effects of CTI on mental health of vulnerable people (Herman et al. 2000; Kasprow and Rosenheck 2007; de Vet et al. 2017a) to a new population. The reduction of PTSD symptoms is of great importance, because of the huge impact of PTSD on the well-being of women and their children (Johnson et al. 2008; Maddoux et al. 2016). Moreover, this study shows a fourfold decrease of unmet care needs in the experimental group compared to the control group. This is a very important reduction, knowing that meeting the service needs of women is key to a successful transition to community living (Ham-Rowbottom et al. 2005; Sullivan et al. 1992). Previous research in Dutch women’s shelters showed that non-native Dutch women had significantly more unmet care needs than native Dutch women (Wolf et al. 2006). In this study, the effect of CTI seems to a large degree to be related to a decline in the proportion Dutch-speaking women with unmet care needs. This finding suggests that it may be more difficult to respond to migrants’ care needs, which corresponds with findings from other studies showing that migrants face several barriers in access to health services, such as restricted legal entitlements and (in)direct discrimination (Rechel et al. 2013).

Three general reasons may explain the lack of effect of CTI on the primary outcome and several other outcomes. First, CTI was fairly implemented in the participating organizations (de Vet et al. 2017b). Research shows that high-fidelity programs produce better client outcomes (Fukui et al. 2012; McHugo et al. 1999). Better outcomes may have been obtained in the experimental group if CTI had been delivered with higher fidelity to the model.

Second, just before the start of the study, the shelter services were professionalized by implementing a strengths-based approach (Wolf and Jansen 2011). The organizations reported that they also employed the strengths-based approach in their services after shelter exit in the control group. As CTI also has elements of the strengths-based approach, this may have led to women in the CTI and care-as-usual group receiving help that was similar in certain respects. The strengths-based approach is geared towards improving the quality of daily lives of people, which may explain the lack of effect of CTI on the primary outcome. This lack of effect may also be explained by the quantity of services provided in both groups. The process measures showed that substantial post-discharge services were delivered to the vast majority of the control group, thereby potentially obscuring the impact of post-discharge case management which is the key element in CTI.

Finally, the present study focused on effects of CTI during the 9-month intervention, whereas in a previous study of CTI the difference in the primary outcome in favor of the experimental group became apparent after more than 12 months (Herman et al. 2011). A follow-up period up to 2 years may be needed to determine the full long-term effects of CTI.

A similar Dutch study showed that homeless people who received CTI during their transition to the community experienced more family support and those with little social support experienced less psychological distress (de Vet et al. 2017a). In this study, no effect was found for family or social support. An important consideration is that abused women by definition have serious problems in their network and often need to move to another community. It is as yet unclear what particular interventions are needed to re-establish their social network.

### Strengths and limitations

This study is unique, as RCTs on interventions for abused women who move from women’s shelters into the community are scarce. The drop-out of this study was very low (6%) and we could include all women in the analysis of the primary outcome. We consider it a strength that non-Dutch-speaking women were included using multilingual interviewers and interpreters. A shortcoming, however, is that for these women more data were missing due to the necessary shortening of the questionnaire. A suggestion for future research in Dutch women’s shelters is to stratify by mastery of language.

Another point of consideration is that the results of this study should be interpreted with some caution as they showed some patterns that could not be explained easily, such as a non-significant effect in the opposite direction of what was expected for the secondary outcome depression.

Further inspection of the scores of Dutch-speaking and non-Dutch-speaking women separately on the significant outcomes of CTI in this study revealed a different effect of CTI on these groups. More research with a larger number of non-Dutch-speaking women needs to been done to confirm this finding. Future research should also focus on the vulnerable position of migrants and what is necessary for interventions to be successful for this particular population.

## Conclusion

This study shows that CTI is applicable not only in the US or to homeless or mentally ill people, but also in another country, with another health care system, and to abused women. CTI has the potential to significantly impact the lives of abused women and their children by reducing symptoms of PTSD and unmet care needs. Given the problems these women face, CTI is an intervention that may help to turn their lives around for the better.

## Electronic supplementary material

Below is the link to the electronic supplementary material.

Supplementary material 1 (DOCX 41 kb)

## Footnotes

1 Honor-related violence is prevalent in communities with a group culture, such as cultures around the Mediterranean Sea and the Middle East. In the Netherlands, it also occurs among Roma, Hindu and orthodox Christian communities (Movisie 2011). Specific examples of honor-related violence are violence conducted to women who refuse an arranged marriage, women who filed for a divorce or women who behave too ‘Western’ according to family members.
