# Supplementary material for: The effectiveness of critical time intervention for abused women leaving women’s shelters: a randomized controlled trial
Source: Int J Public Health. 2018 Jan 3;63(4):513–23. doi: 10.1007/s00038-017-1067-1 (PMC5938300; doi:10.1007/s00038-017-1067-1)
Supplement: Supplementary file 1 — Supplementary material 1 (DOCX 41 kb) [file 38_2017_1067_MOESM1_ESM.docx]

International Journal of Public Health

The effectiveness of critical time intervention for abused women leaving women’s shelters: A randomized controlled trial.

Lako DAM, Beijersbergen MD, Jonker IE, de Vet R, Herman DB, van Hemert AM, Wolf JRLM. Corresponding author: Judith Wolf (Impuls - Netherlands Center for Social Care Research, Department of Primary and Community Care, Radboud university medical center), judith.wolf@radboudumc.nl

Appendix 1. Critical time intervention (CTI) training

The case managers were required to have a higher vocational education and broad experience in working with abused women in shelters. They followed a three-day training in CTI provided by an experienced trainer. The training consisted of the intervention’s theoretical and procedural aspects and practical skills (e.g., motivational interviewing). Each organization was required to assign an internal coach who had to coordinate the CTI work and monitor the model fidelity of the intervention. For this purpose, the coach and the CTI workers had biweekly face-to-face supervision. The coaches received a one-day CTI training at the start of the study and four half-day training sessions during the study period. The researchers organized centralized training sessions for the CTI workers (bi)monthly during the first year and quarterly during the second year of the study. During these training sessions the research team and the trainer offered workshops on how to use CTI chart forms as tools for clients’ care; facilitated discussions in which CTI workers exchanged experiences; and invited CTI experts to present methods for enhancing CTI model fidelity.

Appendix 2. Key components of CTI and process measures

According to the CTI Fidelity Scale Manual^[[1]](#footnote-1)^, the 14 key components of CTI are:

1. Small caseloads, composed exclusively of CTI clients
2. A time-limited, 9-month intervention
3. Decreasing intensity of services
4. Three distinct treatment phases
5. In vivo (i.e., community-based) needs assessment and provision of services
6. Early establishment of community linkages
7. A focus on 1-3 areas that put client at risk for homelessness, selected from the six CTI areas
8. Strengthening of community linkages through negotiation and mediation
9. Worker availability to clients and providers from the field
10. Worker-client relationship characterised by social solidarity
11. Maintaining contact with clients with histories of transience, in order to minimize drop outs
12. A harm-reduction approach to behaviour change
13. Regular team supervision meetings and frequent case review for every CTI client
14. Organizational advocacy, basic staffing and resources, structural flexibility for the CTI program

Six of these key components (#2, #3, #5, #6, #8, #11) were translated into process measures (see figure A1) and administered at the 3-month, 6-month, and 9-month follow-up. Descriptions of the key components were derived from the CTI fidelity scale^[[2]](#footnote-2)^.

**Fig A1.** Translation of key components of CTI (#2, #3, #5, #6, #8, #11) into process measures (The Netherlands, 2010-2013)

| Key component | Description | Process measure(s) |
| --- | --- | --- |
| 2. A time-limited, 9-month intervention | The CTI worker should still be in touch with the client and be providing intervention up to the time of the 9-month post-discharge due date. | *Did you receive support services from the shelter organization since the last interview?* |
| 3. Decreasing intensity of services | CTI involves intensive outreach during Phase 1, then gradually decreases in intensity until Phase 3 when CTI is about monitoring. | *How often did you meet your CTI worker/case manager in the past three months?* |
|  |  | *How often did you talk to your CTI worker/case manager on the phone in the past three months?* |
| 5. In vivo (i.e., community-based) needs assessment and provision of services | Preferably, the CTI worker should not provide shelter-based standard case management in addition to CTI. | *Is your CTI worker/case manager the same person who provided services to you in the shelter? Or is this someone else?* (3-month follow-up only) |
|  | The CTI worker should visit the client where he/she is living or receiving community services. | *Where did you usually meet your CTI worker/case manager?* |
| 6. Early establishment of community linkages | The CTI worker should meet at least once a month with the client before discharge. | *Did you already meet or talk to your CTI worker/case manager during shelter stay?* (3-month follow-up only) |
| 8. Strengthening of community linkages through negotiation and mediation | The CTI worker should encourage communication between the client and community linkages. | *Do you receive help from other professionals or services to achieve your goals?* |
| 11. Maintaining contact with clients with histories of transience, in order to minimize drop outs | The CTI worker should have provided at least seven months of active post-discharge intervention. | *How often did you meet your CTI worker/case manager in the past three months?* |
|  |  | *How often did you talk to your CTI worker/case manager on the phone in the past three months?* |

Appendix 3. Outcome measures excluded from this work

In the study protocol we described another six outcome measures that we measured in the study: loneliness, substance use, service use, working alliance between women and CTI workers or case managers, experiences with shelter and community care services and parenting stress. All outcome measures were assessed at baseline and 9-month follow-up. The outcome measures and the reasons for excluding them from this work are described below. To identify the most important outcome measures we also consulted the literature for similar studies.

Loneliness was measured with the De Jong Gierveld and Kamphuis Loneliness Scale (de Jong-Gierveld and Kamphuis 1985). Loneliness and family and social support measure to some extent the same concept. Because CTI aims to connect clients with sources of support, we expected the measures family and social support to be more sensitive to intervention effects than the multidimensional construct of loneliness (DiTommaso and Spinner 1997).

Substance use was measured with the European version of the Addiction Severity Index (EuropASI) (McLellan et al. 1992; Kokkevi et al. 1993). Because few women used alcohol to the level of intoxication (≥ 5 drinks), cannabis, or any other substances in the past 30 days at both time points, adequate statistical comparison was not possible.

Service use was measured with a self-constructed instrument used in several studies by Impuls - Netherlands Center for Social Care Research. Women were asked to indicate whether they had used the services of certain care providers (e.g., general practitioner, dentist, social services) in the past nine months and in the past 30 days. Unfortunately, this instrument was not correctly used in the questionnaire which made women with missing values indistinguishable from women who did not use specific services in a certain period. Therefore, the answers of this questionnaire were not usable.

Working alliance between women and CTI workers or case managers was measured with the short version of the Working Alliance Inventory (WAI) (Horvath and Greenberg 1986; Vervaeke and Vertommen 1996). This instrument was administrated only to the women who indicated receiving services from a shelter organization at the 9-month follow-up. Because fewer women in the control group still received services from a shelter organization at 9-month follow-up, considerably more observations were missing from this group, which could result in biased estimates.

Experiences with shelter and community care services were assessed with the Consumer Quality Index for Shelter and Community Care Services (CQI-SCCS) (Beijersbergen et al. 2015). Unfortunately, missing data on this instrument was high and given the conceptual overlap with the process measures and the outcome unmet care needs, we choose not to include the outcome measure in this work.

Parenting stress was measured with two scales of the experimental version of the Parenting Stress Questionnaire (Vermulst et al. 2012). By answering the scale items, the parent has to keep one child in mind. Unfortunately, this instruction was not given to the interviewers and the women answered the items for all children (if they had more than one child). Therefore, the data collected with this instrument was not reliable.

Appendix 4. Missing data

Due to the use of a shortened version of the questionnaire for women who did not speak Dutch, a substantial proportion of women had missing data on the secondary and intermediate outcomes at baseline and 9-month follow-up (range: 4-13%). More than 10% of the women were excluded from the analyses of psychological distress, self-esteem (secondary outcomes), family support, and social support (intermediate outcomes).

The proportion of missing values was higher in the control condition than in the experimental condition for all four outcome measures: 17% versus 7% for the outcome psychological distress, 15% versus 7% for the outcome self-esteem, and 18% versus 7% for the outcomes family support and social support, but the differences were not statistically significant (*p* > .05). Women who were excluded were mostly first-generation migrants (*p* < .05) and they experienced sexual violence less often before shelter entrance although not significantly (*p* > .05). Excluded women did not differ from women with observed values on other sociodemographic characteristics (*p* > .05).

Re-abuse was assessed at 3-month, 6-month and 9-month follow-up using the question *Have you been abused since the last interview?* The date of the last interview was added to this question to make sure that the 9-month study period was covered. However, in some cases we did not acquire complete information, for example if women had a missing value on 3-month follow-up and reported re-abuse at 6-month follow-up, we did not ask whether the re-abuse was experienced before or after 3-month follow-up. For this reason, we could not include two women who had a missing value on 3-month follow-up and reported re-abuse at 6-month follow-up and two women who had missing values on 3-month and 6-month follow-up and reported re-abuse at 9-month follow-up. Furthermore, two women could not be reached after the baseline interview and seven others had missing values at 9-month follow-up, and were therefore not included in the analysis. In total, 13 women (10%) were not included in the analysis of the outcome re-abuse.

Appendix 5.

Table A1 Process measures in experimental (critical time intervention) and control (care-as-usual) group at 3-month, 6-month, and 9-month follow-up (The Netherlands, 2010-2013)^a^

|  | 3-month follow-up | | 6-month follow-up | | 9-month follow-up | |
| --- | --- | --- | --- | --- | --- | --- |
|  | CTI  (*n* = 70) | CAU  (*n* = 66) | CTI  (*n* = 70) | CAU  (*n* = 66) | CTI  (*n* = 70) | CAU  (*n* = 66) |
| All women | *n* = 64 | *n* = 57 | *n* = 62 | *n* = 58 | *n* = 56 | *n* = 51 |
| Services received from CTI worker/case manager | 61 (95%) | 49 (86%) | 59 (95%) | 38 (66%) | 48 (86%) | 31 (61%) |
|  |  |  |  |  |  |  |
| Frequency of face-to-face contact^b^ | *n* = 58 | *n* = 55 | *n* = 60 | *n* = 58 | *n* = 47 | *n* = 46 |
| Not at all | 4 (7%) | 8 (15%) | 4 (7%) | 24 (41%) | 9 (19%) | 25 (54%) |
| Less than once a month | 3 (5%) | 6 (11%) | 2 (3%) | 5 (9%) | 2 (4%) | 4 (9%) |
| At least once a month | 18 (31%) | 20 (36%) | 29 (48%) | 15 (26%) | 23 (49%) | 9 (20%) |
| At least once a week | 33 (57%) | 21 (38%) | 25 (42%) | 14 (24%) | 13 (28%) | 8 (17%) |
|  |  |  |  |  |  |  |
| Frequency of telephone contact^b^ | *n* = 56 | *n* = 55 | *n* = 60 | *n* = 58 | *n* = 47 | *n* = 46 |
| Not at all | 9 (16%) | 15 (27%) | 13 (22%) | 26 (45%) | 12 (26%) | 27 (59%) |
| Less than once a month | 13 (23%) | 16 (29%) | 16 (27%) | 9 (16%) | 14 (30%) | 4 (9%) |
| At least once a month | 19 (34%) | 16 (29%) | 19 (32%) | 17 (29%) | 12 (26%) | 9 (20%) |
| At least once a week | 15 (27%) | 8 (15%) | 12 (20%) | 6 (10%) | 9 (19%) | 6 (13%) |
|  |  |  |  |  |  |  |
| Women who received services only |  |  |  |  |  |  |
| Most frequent location of face-to-face contact | *n* = 54 | *n* = 47 | *n* = 56 | *n* = 34 | *n* = 38 | *n* = 21 |
| Woman’s house | 52 (96%) | 35 (75%) | 55 (98%) | 26 (77%) | 36 (95%) | 17 (81%) |
| CTI worker’s/case manager’s office | 1 (2%) | 8 (17%) | 1 (2%) | 8 (24%) | 1 (3%) | 3 (14%) |
| Shelter | 1 (2%) | 4 (9%) | 0 (0%) | 0 (0%) | 1 (3%) | 1 (5%) |
|  |  |  |  |  |  |  |
| Help from other professionals and service agencies | *n* = 54 | *n* = 46 | *n* = 57 | *n* = 38 | *n* = 40 | *n* = 24 |
| Never | 17 (32%) | 16 (35%) | 14 (25%) | 8 (21%) | 8 (20%) | 5 (21%) |
| Sometimes | 16 (30%) | 15 (33%) | 18 (32%) | 13 (34%) | 10 (25%) | 7 (29%) |
| Often | 9 (17%) | 6 (13%) | 15 (26%) | 12 (32%) | 10 (25%) | 10 (42%) |
| Always | 12 (22%) | 9 (20%) | 10 (18%) | 5 (13%) | 12 (30%) | 2 (8%) |

^a^ *CTI* critical time intervention, *CAU* care-as-usual

^b^ Between women and CTI worker/case manager in past 3 months

References

Beijersbergen MD, Asmoredjo JK, Christians MGM, Wolf JRLM (2015) Psychometric properties of the consumer quality index to assess shelter and community care services. Eur J Public Health 25:378-384. doi:10.1093/eurpub/cku195

de Jong-Gierveld J, Kamphuis F (1985) The development of a Rasch-type loneliness scale. Appl Psych Meas 9:289-299

DiTommaso E, Spinner B (1997) Social and emotional loneliness: a re-examination of Weiss' typology of loneliness. Pers Indiv Differ 22:417-427. doi:10.1016/S0191-8869(96)00204-8

Horvath AO, Greenberg LS (1986) The development of the working alliance inventory. In: Greenberg LS, Pinsof WM (eds) The psychotherapeutic process: a research handbook. Guilford Press, New York, pp 529-556

Kokkevi A, Hartgers C, Blanken P, Fahrner EM, Pozzi G, Tempesta E, et al. (1993) European version of the addiction severity index. 5th edn. [Dutch translation by Hartgers C, Hendriks V, van der Meer CW, Blanken P (1994)]. Amsterdams Instituut voor Verslavings Onderzoek, Amsterdam

McLellan AT, Kushner H, Metzger D, Peters R, Smith I, Grissom G, et al. (1992) The fifth edition of the addiction severity index. J Subst Abuse Treat 9:199-213. doi:10.1016/0740-5472(92)90062-S

Vermulst A, Kroes G, De Meyer R, Nguyen L, Veerman JW (2012) Opvoedingsbelastingvragenlijst (OBVL). Handleiding [Parenting Stress Questionnaire. Manual]. Praktikon, Nijmegen

Vervaeke GAC, Vertommen H (1996) Kort instrumenteel, de Werkalliantievragenlijst [Measurement instruments, the Working Alliance Inventory]. Gedragstherapie 29:139-144

1. Conover S (2012) CTI fidelity scale manual. Department of Psychiatry, Columbia University, New York (unpublished) [↑](#footnote-ref-1)
2. Conover S, Herman DB (2007) CTI fidelity scale. Department of Epidemiology and the Center for Homelessness Prevention Studies, Columbia University Mailman School of Public Health, New York (unpublished) [↑](#footnote-ref-2)
